# Effectiveness and Safety of Mitomycin C in Deep Sclerectomy with the Esnoper Clip Implant: A Case Series Study

**DOI:** 10.3390/jcm14176085

**Published:** 2025-08-28

**Authors:** Rachid Bouchikh-El Jarroudi, Alina-Dana Baxant, Pavel Studeny, Kolbe Roche Fernández, Adrian Sanchez-Fortun-Sanchez, Martin Pencak, Jessica Botella-Garcia, Jordi Loscos-Arenas, Antoni Sabala Llopart, Sebastian Videla, Pau Romera-Romero

**Affiliations:** 1 Service of Ophthalmology, Germans Trias i Pujol University Hospital, 08916 Badalona, Spain; 2 Department of Surgery, Barcelona Autonomous University (UAB), 08035 Barcelona, Spain; 3 Evidence Based Ophthalmology Unit (Oftalmoevidencia), Scientia Clinical and Epidemiological Research Institute, Trujillo 13001, Peru; 4 Service of Ophthalmology, University Hospital Kralovske Vinohrady, 10000 Prague, Czech Republic; 5 Clinical Research Support Area, Clinical Pharmacology Department, Hospital Universitari Germans Trias i Pujol, 08916 Badalona, Spain; 6 Department of Pharmacology, Therapeutics and Toxicology, Autonomous University of Barcelona, 08193 Bellaterra, Spain

**Keywords:** glaucoma, Deep Sclerectomy, Esnoper Clip, mitomycin C, fibrosis, safety

## Abstract

**Objective**: The objective was to provide evidence on the effectiveness (intraocular pressure, IOP) and safety of Deep Sclerectomy with the Esnoper Clip implant with and without the use of mitomycin C (MMC) in uncontrolled primary open-angle glaucoma. **Methods**: This was a multicentric, retrospective, consecutive case series study undertaken as a collaboration between University Hospital Trias i Pujol, Spain, and University Hospital Kralovske Vinohrady, Czech Republic. The IOP reduction was compared between patients who underwent Deep Sclerectomy with the Esnoper Clip implant, where the antifibrotic agent mitomycin C was used perioperatively (MMC group), and patients who underwent Deep Sclerectomy with Esnoper Clip implants where mitomycin C was not used either peri- or postoperatively (non-MMC group). At the end of the study (12 months after surgery), complete success probability (defined as an IOP <21 mmHg without medication) and qualified success probability (defined as an IOP <21 mmHg with or without medication) were assessed. Safety was based on postoperative complications. A descriptive and exploratory analysis was performed. **Results**: Between January 2017 and June 2019, a total of 72 consecutive patients (40 women and 32 men; age [median (range)] 70 (39–90)) with uncontrolled primary open-angle glaucoma who underwent Deep Sclerectomy with an Esnoper Clip implant were included. Of these, 78 eyes were included: 44 eyes (38 patients) in the MMC group and 34 eyes (34 patients) in the non-MMC group. At the end of the study (38 and 32 eyes, respectively), the complete success probabilities (95% confidence interval) were 62.2% (51.5–75.2%) and 80.5% (69.5–93.2%), *p*-value: 0.001; qualified success probability (95% confidence interval): 84.7% (76.6–93.7%) and 85.7% (75.8–97%), *p*-value: 0.188; and complications (total): 36 and 24, *p*-value: 0.286. **Conclusions**: In this case series study, the use of mitomycin C in Deep Sclerectomy with the Esnoper Clip implant for patients with uncontrolled primary open-angle glaucoma does not appear to improve IOP one year after surgery. No safety issues related to mitomycin C were observed. Further study is needed to confirm the effectiveness and to assess long-term safety.

## 1. Introduction

Glaucoma, the leading cause of irreversible blindness worldwide [1], is a progressive optic neuropathy [2,3] associated with various risk factors, including elevated intraocular pressure (IOP) [4], older age, and family history of glaucoma [5], among others [2]. At present, IOP remains the only modifiable risk factor, and thus, treatment is focused on this variable [6,7] in order to delay the progression of glaucoma by decreasing IOP [8].

Surgery is indicated when medication or laser treatment to lower IOP proves insufficient [2,9]. Deep Sclerectomy (DS) is a glaucoma surgery that aims to enhance aqueous humor outflow and reduce IOP without opening the anterior chamber, creating a trabeculo-descemetic window [10,11].

The use of hydrophilic implants under the scleral flap to maintain the subscleral space is an accepted method used worldwide, and numerous reports highlight their benefits [10,11,12,13,14]. The Esnoper Clip (EC) (AJL, Alava, Spain) is a hydroxyethyl methacrylate (HEMA) implant characterized by a hinge with two plates, with one located in the suprachoroidal space and the other in the intrascleral space. Its safety has been widely demonstrated [11,14,15,16].

One of the outstanding problems with the DS technique, as with other glaucoma surgeries, is the healing process, which causes subconjunctival fibrosis and adhesions in the intrascleral filtrating space, both of which obstruct the filtration site [17]. To mitigate this problem, mitomycin C (MMC), a potent antifibrotic agent, has been used as an adjunct to glaucoma surgery. MMC is an alkylation agent targeting DNA, inhibiting fibroblast proliferation and, consequently, decreasing collagen synthesis in the extracellular matrix. This, in turn, reduces the risk of the DS failure [18]. In several studies, MMC has provided benefits for DS patients, such as reducing postoperative scarring, decreasing postoperative IOP, and improving surgical success rates [10,18,19,20,21,22]. However, MMC has not always been associated with better control of IOP [23], and its application also has undesirable effects, for instance, avascular blebs and conjunctival oozing [24].

Our working hypothesis was that, in patients with uncontrolled primary open-angle glaucoma, the use of MMC in Deep Sclerectomy with the Esnoper Clip implant (DS-with-EC) improves the effectiveness of this glaucoma surgery. Therefore, the aim of this consecutive case series study was to provide evidence on the effectiveness (reduction in IOP) and safety of DS-with-EC with and without MMC.

## 2. Materials and Methods

### 2.1. Study Design

This study was a multicentric, retrospective, consecutive case series study undertaken as a collaboration between University Hospital Trias i Pujol, Spain (“center 1”), and University Hospital Kralovske Vinohrady, Czech Republic (“center 2”).

This study complied with the updated Declaration of Helsinki, Good Clinical Practice guidelines, and applicable Spanish and European regulatory requirements. Confidentiality was ensured following the European Regulation (EU) 2016/679 of the European Parliament and Council, and current Spanish law (LOPD 3/2018). This manuscript complies with the STROBE statement.

### 2.2. Study Population

Patients who underwent DS-with-EC implants from a center where MMC as an antifibrotic agent was used perioperatively (MMC group, “center 1”) and patients who underwent DS-with-EC implants from a center where MMC was not used either peri- or postoperatively (non-MMC group, “center 2”) as standard clinical practice at each center were retrospectively analyzed.

The inclusion criteria were (i) all adult patients (≥18 years of age) of either sex, (ii) Caucasian, (iii) with a diagnosis of uncontrolled primary open-angle glaucoma (POAG), defined as an IOP >21 or glaucoma progression in the visual field or on optical coherence tomography despite pharmacological treatment (maximum tolerated therapy), (iv) who underwent surgical intervention, (v) without previous glaucoma surgery, (vi) with clinical data available in the medical file, and (vii) who signed informed consent accepting that their medical history data could be used for research purposes.

The starting point of this study was January 2017, when “center 2” began performing DS-with-EC. Therefore, at that time, both centers were treating uncontrolled POAG with the same technique. Then, consecutive sampling was performed, including all patients who met the inclusion criteria between January 2017 and June 2019.

### 2.3. Surgical Technique

Surgical interventions were performed by one designated surgeon in each hospital (P.R.R. at “center 1” (MMC group) and P.S. at “center 2” (non-MMC group).

**Figure 1:**
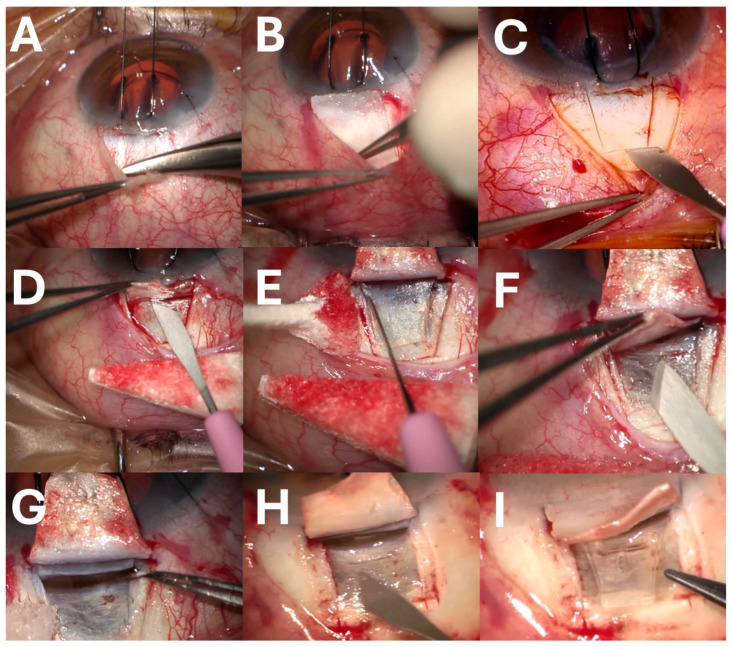
Image of the Deep Sclerectomy technique step by step (**A**). The preparation of a fornix-based conjunctival flap. (**B**). A sheet impregnated with mitomycin C is laid under the posterior conjunctiva and left in place for 2 min. The preparation (**C**) and dissection (**D**) of the superficial scleral flap. Dissection of the deeper scleral flap (**E**) and its removal (**F**). (**G**). The Schlemm’s canal deroofing with capsulorhexis forceps. (**H**). The suprachoroidal pocket preparation. (**I**). The Esnoper Clip implantation in the suprachoroidal pocket.

In “center 1” (MMC group) (Figure 1), a superior 6/0 Nylon traction suture was passed through the superior cornea, and a fornix-based conjunctival flap was dissected, followed by cauterization of bleeding vessels and dissection of the superficial scleral flap (4 × 5 mm) to one-third of the scleral depth, extending 2 mm into the clear cornea. Afterward, an MMC-impregnated sheet at 0.02% was laid under the posterior conjunctiva and left in place for 2 min. The area was then irrigated with a 20 mL balanced salt solution. Subsequently, a deeper 3 × 4 mm scleral flap was dissected and removed, and the Schlemm canal was deroofed with capsulorhexis forceps. The EC implant was then placed in a suprachoroidal pocket, and the superficial flap was sutured loosely with Nylon 10/0. Finally, the conjunctiva was sutured with a Nylon 10/0 and the sutures were extracted 2 weeks after surgery. Postoperative treatment consisted of topical ciprofloxacin (3 mg/mL) 3 times daily for 1 week and dexamethasone (1 mg/mL) initially 6 times daily and in a descending dosage over 3 months.

In “center 2” (non-MMC group), the conjunctiva was opened 4 mm from the limbus and a Silk 4/0 suture was used to fix the prepared conjunctiva on the eyelid speculum. Then, the dissection of the superficial and deep scleral flaps in a square shape was carried out. The preparation of a superficial scleral lamella of 4 × 4 mm at a depth of approximately one-third of the scleral thickness followed. The superficial scleral dissection was extended 1.5–2 mm through the limbus into the clear cornea. The preparation and resection of a deep scleral lamella of 3 × 3 mm followed. Then, the Schlemm canal endothelium was peeled off with capsular forceps. Further, 1.5–2 mm behind the scleral spur, a suprachoroidal pocket was created. The EC implant was then placed in the suprachoroidal pocket, and the superficial flap was then sutured with Vicryl 8/0. Finally, the conjunctiva was sutured with Vicryl 8/0. None of the patients in this group received MMC, nor other antimetabolites or a wound healing modulator, such as a collagen matrix implant, during or after surgery. Postoperatively, all patients were prescribed a suspension of topical antibiotics and steroids (3 mg/mL tobramycin, 1 mg/mL dexamethasone) 5 times daily for 2 weeks and cycloplegics (4% homatropine hydrobromide) when necessary. Then, therapy was continued through mild steroids (0.1% fluorometholone acetate) for 10 additional weeks.

In both groups, goniopuncture (GP) with the YAG laser was performed when percolation of the aqueous humor through the trabeculodescemetic membrane was considered insufficient, or in cases of glaucoma progression, or when the individual IOP target was not reached. Furthermore, 27-gauge needle revision was carried out when blebs became flattened and the IOP exceeded objective limits.

### 2.4. Endpoints

The primary outcome was the complete success probability at 12 months after surgery, defined as IOP < 21 mmHg without medication [17].

The secondary outcomes were as follows:

- Qualified success probability at 12 months after surgery, defined as an IOP < 21 mmHg with or without medication [17,22].
- “Failure of the glaucoma surgery” during the first year of follow-up, defined as an IOP > 21 or <6 mmHg confirmed at 2 consecutive follow-ups, any additional glaucoma surgery required, and/or loss of light perception, based on the Guidelines on Design and Reporting of Glaucoma Surgical Trials [25].
- IOP during the follow-up measured with the Goldmann Applanation Tonometer (mmHg), IOP reduction (difference in IOP compared with the baseline, mmHg) and IOP reduction percentage (difference in IOP compared with the baseline, %), and recorded baseline (preoperatively, defined as the IOP taken at the last preoperative visit) at 1 day, 1 week, and 1, 3, 6, and 12 months after surgery.
- The number of GPs, needlings, and glaucoma medications (eye-pressure-lowering medication) required were also recorded.

The safety outcome was assessed by the number of postoperative complications.

### 2.5. Statistical Analysis

Given the exploratory nature of this study, no formal sample size calculation was conducted. The final sample size analyzed included all eligible patients within the specified time frame (i.e., a convenience sample).

Categorical variables were expressed as absolute and relative frequencies. Quantitative variables were expressed as medians and ranges or as means and standard deviations (SD), as applicable. A descriptive and exploratory analysis between the MMC group and the non-MMC group was performed. The “complete success” and “qualified success” at 12 months were estimated, and their 95% confidence interval (95% CI) was calculated. Kaplan–Meier plots were generated for obtaining survival data. The Log-Rank test was used to compare the two groups.

The distribution normality of measurements was assessed using the Shapiro–Wilk test. Being the non-normal distribution of all variables (non-parametric), the Mann–Whitney U test was used to compare the MMC group and the non-MMC group (IOP, IOP decrease, IOP percentage decrease, and medication). Dichotomous variables were analyzed with χ^2^, and Fisher’s exact test if any cell value counts in the contingency table were less than 5. The statistical significance level was set at *p* < 0.05. Data analysis was performed using Jamovi software version 2.1 (https://www.jamovi.org (accessed on 15 March 2025)).

## 3. Results

### 3.1. Baseline Characteristics

Between January 2017 and June 2019, a total of 72 consecutive patients (40 women and 32 men) with uncontrolled POAG who underwent DS-with-EC were included in the study. Of these, a total of 78 eyes (44 women and 34 men) were included: 44 eyes (38 patients) in the MMC group and 34 eyes (34 patients) in the non-MMC group.

Regarding the indication for surgery, in center 1 (MMC group), 52.3% of eyes (23/44) were included because of uncontrolled IOP >21 mmHg despite maximum tolerated medical therapy, while the remaining 47.7% (21/44) were included due to documented glaucoma progression in the visual field or on optical coherence tomography. In center 2 (non-MMC group), 20.6% of eyes (7/34) were included for an IOP > 21 mmHg, whereas 79.4% (27/34) were included for glaucoma progression despite receiving the maximum tolerated topical treatment.

**Figure 2:**
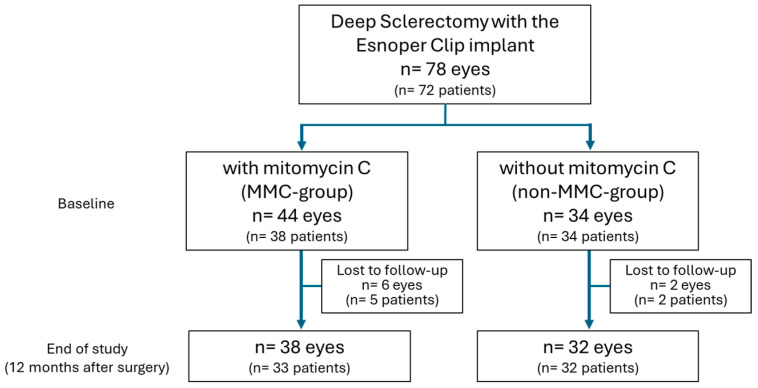
Study population flowchart: patients with a diagnosis of uncontrolled primary open-angle glaucoma or glaucoma progression in the visual field or on optical coherence tomography despite pharmacological treatment (maximum tolerated therapy).

The baseline characteristics are shown in Table 1. The IOP was higher at baseline in the MMC group. Figure 2 shows the flow chart.

There were no differences in the number of glaucoma medications between the MMC group and the non-MMC group at baseline and during the follow-up period. The MMC group required more GP than the non-MMC group [23 (52.3%) and 11 (32.4%), respectively; *p*-value: 0.079], and more needling during the follow-up period [5 (11.4%) and 0 (0%), respectively; *p*-value: 0.065].

### 3.2. Effectiveness of MMC

**Figure 3:**
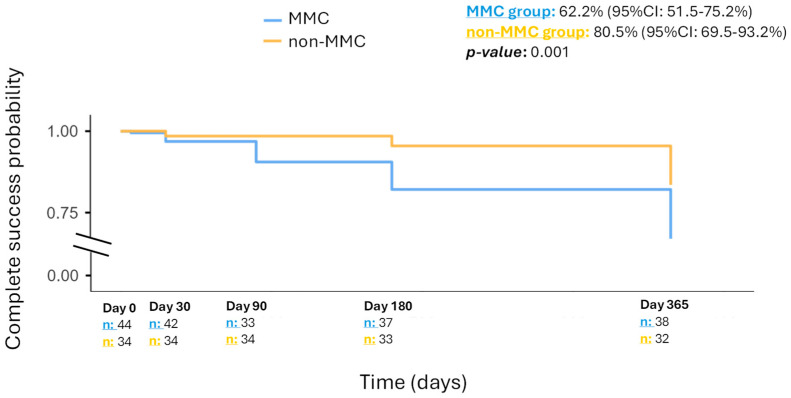
Kaplan–Meier curve displaying the complete success probability in each group, respectively.

At the end of the study (12 months after), data was available for 38 eyes in the MMC group and 32 eyes in the non-MMC group. The complete success probability at 12 months was 62.2% (95%CI: 51.5–75.2%) in the MMC group and 80.5% (95%CI: 69.5–93.2%) in the non-MMC group (*p*-value: 0.001). Figure 3 shows the Kaplan–Meier curve for the estimated probability of complete success.

**Figure 4:**
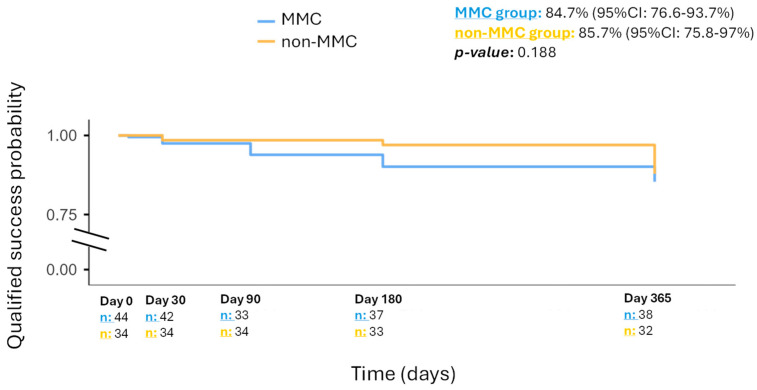
Kaplan–Meier curve displaying the qualified success probability in each group, respectively.

The qualified success probability at 12 months was 84.7% (95%CI: 76.6–93.7%) in the MMC group and 85.7% (95%CI: 75.8–97%) in the non-MMC group (*p*-value: 0.188). Figure 4 shows the Kaplan–Meier curve for the estimated probability of qualified success.

Table 2 presents the failures of DS-with-EC at 1 year of follow-up, with no differences between groups.

**Figure 5:**
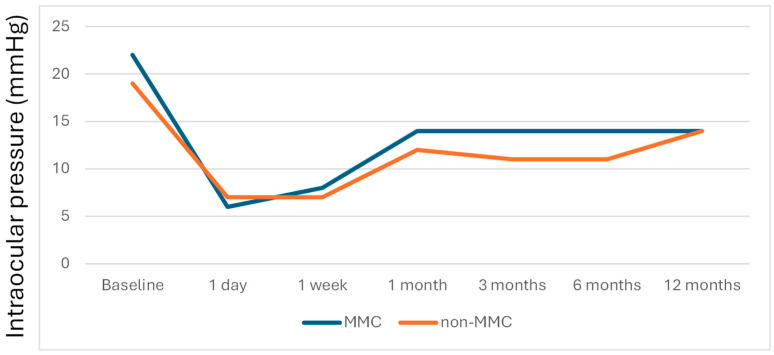
Evolution of median IOP (mmHg) in both studied groups.

**Figure 6:**
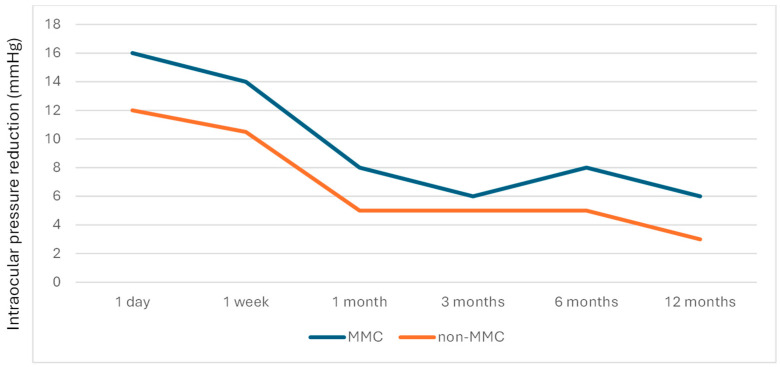
Evolution of the difference (baseline–follow-up point) median IOP (mmHg) in both studied groups.

**Figure 7:**
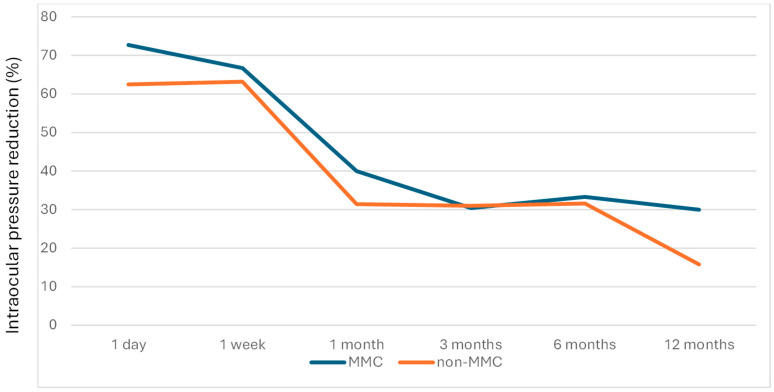
Evolution of IOP (mmHg), based on the percentage IOP change with respect to baseline, in both studied groups.

Table 3 presents the IOP, IOP reduction, and medication across time in the MMC group and the non-MMC group, and Figure 5, Figure 6 and Figure 7 depict the linear graphs for these results. No differences in the IOP during the follow-up were detected. It is noteworthy that, as mentioned above, the preoperative IOP was greater in the MMC group. In the MMC group, the IOP reduction was significantly higher at 1 day, 1 week, and 1 year. Likewise, the percentage of IOP reduction in the MMC group was significantly higher at 1 day and 1 year. A sensitivity analysis was performed, so that patients who underwent surgery on both eyes were only included in this analysis for the first eye operated on, and no changes in the results were observed.

### 3.3. Safety

Table 4 represents the complications after DS-with-EC, with no differences between groups, except for choroidal detachment in the early postoperative period, which was more frequent in the non-MMC group [0 (0%) in the MMC group and 6 (17.6%) in the non-MMC group; *p*-value 0.005].

## 4. Discussion

To the best of our knowledge, this study is the first to provide evidence on the effectiveness and safety of DS-with-EC with and without MMC. In fact, this study provides evidence on the possible synergistic effect of the EC implant and mitomycin C, added to DS, in patients with uncontrolled POAG. The results of this case series study do not support our working hypothesis: “The use of mitomycin C in Deep Sclerectomy with the Esnoper Clip implant to treat patients with uncontrolled primary open-angle glaucoma improves the effectiveness of this glaucoma surgery”. The IOP decreased in both groups, mainly in patients who received MMC on the first day after surgery. Nevertheless, complete success (defined as an IOP < 21 mmHg without medication at the end of the study) was greater in the non-MMC group. No differences in qualified success (defined as IOP < 21 mmHg with or without medication) were found between the MMC group and the non-MMC group.

The efficacy of the perioperative application of MMC during DS was demonstrated in different studies [17,23,24,26,27]. Several randomized clinical trials concluded that the application of intraoperative MMC for DS decreases the IOP more than in patients who have undergone DS surgery without MMC [17,23,26]. Similarly, a retrospective case series study, which evaluated patients who had undergone SD surgery, also showed a greater reduction in IOP in patients in whom MMC was used [24]. Another retrospective study comparing DS-with-phacoemulsification with and without MMC showed a greater IOP decrease in the MMC group [27]. A study in viscocanalostomy concluded that the administration of MMC decreases the IOP more than in patients subjected to nonpenetrating glaucoma surgeries without MMC [22]. The greater reduction in IOP in patients in whom intraoperative MMC was administered was demonstrated in both normotensive glaucoma [26] and POAG [17,23]. On the other hand, a randomized controlled trial conducted in Nigeria, which compared the efficacy of MMC in patients undergoing DS surgery, concluded that MMC was not effective in African American people. The success rates were low in both studied groups: the MMC group and the control group [23]. In addition, it is noteworthy that differences in the progression and severity of glaucoma in different ethnicities have been described [28].

One possible explanation for the unexpected result in the main variable (complete success) could be that around half of the patients in the MMC group (52.3% of eyes) were included because they had an IOP > 21, whereas in the non-MMC group, the main reason for inclusion in the study was glaucoma progression in the visual field or on optical coherence tomography despite drug treatment (maximum tolerated therapy) (79.4% of eyes). Moreover, another explanation could be that the postoperative target IOP was lower in the MMC group. The MMC group received medication more easily even if an IOP < 21 mmHg was achieved. This would also explain why there was no difference in the qualified success probability between the MMC group and the non-MMC group. Similar results in qualified success rate have been described in other studies [22,23,26]. Only one of the randomized studies found a higher qualified success rate in the group with intraoperative MMC [17].

Unlike in previous publications [22,23,26], in our study, a greater number of patients who underwent DS with MMC received more GPs compared with the non-MMC group [23 patients (52.3%) and 11 patients (32.4%), respectively]. This result could be because GP after DS is not considered a surgical failure at the center where the MMC group was located. Rather, GP is performed routinely at this hospital when the target IOP is not met during any postoperative visit [11].

No differences were found between groups in terms of the rate of complications, except for choroidal detachment, which was more frequent in the non-MMC group (0% vs. 17.6%). However, MMC treatment led to a greater IOP reduction at 24 h after surgery. We have no explanation for this finding.

Classically, the application of MMC has been associated with higher rates of side effects after trabeculectomy [18,29,30]. This increased risk of side effects was not demonstrated in DS with MMC. As previously reported, the theory proposes that DS is a non-penetrating technique that could protect against the complications known to be associated with MMC [17].

Among the strengths of this study, we can mention two. The first is that this study captures what happens in clinical practice. The second strength is the sample size (78 eyes out of 72 patients). Most of the studies involving meta-analysis had a sample size of less than 40 [22]. Conversely, certain limitations must be considered when interpreting the results. The study design (retrospective case series study, non-randomized) could lead to an overestimation or underestimation of the results. For example, baseline differences in IOP could be explained by the fact that the study was not randomized. This difference in IOP baseline may play a role in the effectiveness of MMC treatment. To try to minimize this bias, the decrease in IOP was calculated as both an absolute value and as a percentage. Only Caucasian patients were analyzed. As mentioned above, it is possible that there are racial differences between patients in both centers due to the geographic location of the hospitals. Other limitations to highlight are related to surgery or post-surgery management: (i) the surgical technique is surgeon-dependent. Thus, even though the main steps of the surgery are standardized and shared by both surgeons in the study, there are variations between both techniques that may influence the results, such as the dimensions of the scleral buckle, the type of suture used, and the postoperative treatment. (ii) Different sutures are used. Vicryl sutures induce more cellular reaction and inflammation compared to Nylon sutures [31]. (iii) There is a learning curve. Center 1 (MMC group) started using DS-with-EC two years before center 2 (non-MMC group). Hence, the learning curve may have influenced the results, but an assessment of this was not possible [11,14]. (iv) The difference in GP and needle revision rates could be considered a limitation of this study, because it presents a confounding factor that could bias our results related to IOP reduction and success probability.

## 5. Conclusions

In this case series study, the use of mitomycin C in a Deep Sclerectomy with an Esnoper Clip for patients with uncontrolled primary open-angle glaucoma does not appear to improve IOP one year after surgery. No safety issues related to mitomycin C were identified.

There is insufficient evidence to advise against using MMC in DS-with-EC. Further study is needed to confirm the effectiveness and to assess the long-term safety of MMC in DS-with-EC.

## Figures and Tables

**Table 1:** Demographic data of the patients included in the study.

		MMC Group	non-MMC Group	*p*-Value
**Eyes (n)**		44	34	-
**Laterality (Right/Left)**	**Total (n)**	21/23	16/18	0.953
	**Percentage (%)**	47.7%/52.3%	47.1%/52.7%	
**Sex (Women/Men)**	**Total (n)**	25/19	19/15	0.934
	**Percentage (%)**	56.8%/43.2%	55.9%/44.1%	
**Phakic/pseudophakic**	**Total (n)**	20/24	19/15	0.402
	**Percentage (%)**	45.5%/54.5%	55.9%/44.1%	
**Age**	**Mean (standard deviation)**	71.2 (9.91)	69.4 (10)	0.338
	**Median (range)**	72 (44–90)	69 (39–87)	

**Table 2:** Failures of Deep Sclerectomy at 1 year of follow-up.

Cause of Failure	MMC Group n = 44 Eyes	non-MMC Group n = 34 Eyes	*p*-Value
Hypotony (<6 mmHg)	0 (0%)	0 (0%)	1
Hypertony (>21 mmHg)	2 (4.5%)	1 (2.9%)	1
Reoperation	1 (2.3%)	0 (0%)	0.253
Loss of light perception	1 (2.3%)	0 (0%)	0.253
**Overall**	**4 (9.1%)**	**1 (2.9%)**	**0.380**

**Table 3:** IOP evolution with respect to the preoperative state (baseline).

Time Interval		IOP (mmHg)		IOP (mmHg) Reduction with Respect to Baseline		Percentage IOP Reduction with Respect to Baseline		Medication (Number)	
		Median (Range)	*p*-Value	Median (Range)	*p*-Value	Median (Range)	*p*-Value	Median (Range)	*p*-Value
**Preoperative**	**MMC (n = 44)**	22 (14–76)	<0.001	NA	NA	NA	NA	3 (1–4)	0.718
	**non-MMC (n = 34)**	19 (13–34)		NA		NA		3 (2–4)	
**1 day**	**MMC (n = 41)**	6 (0–14)	0.133	16 (6–30)	0.001	72.7 (33.3–100)	0.008	0 (0–0)	1
	**non-MMC (n = 34)**	7 (2–18)		12 (1–22)		62.5 (7.69–86.4)		0 (0–0)	
**1 week**	**MMC (n = 43)**	8 (1–26)	0.781	14 (−2–68)	0.024	66.7 (−11.1–95.8)	0.358	0 (0–0)	1
	**non-MMC (n = 44)**	7 (2–20)		10.5 (0–23)		63.2 (0–85.7)		0 (0–0)	
**1 month**	**MMC (n = 42)**	14 (6–40)	0.084	8 (−19–64)	0.189	40.0 (−90.5–88.4)	0.471	0 (0–1)	0.372
	**non-MMC (n = 34)**	12 (7–27)		5 (−9–22)		31.4 (−64.3–70.8)		0 (0–0)	
**3 months**	**MMC (n = 33)**	14 (8–30)	0.012	6 (−2–24)	0.930	30.4 (−11.1–75.0)	0.629	0 (0–1)	0.074
	**non-MMC (n = 34)**	11 (8–20)		5 (−1–22)		31.9 (−5.88–65.2)		0 (0–0)	
**6 months**	**MMC (n = 37)**	14 (8–35)	0.053	8 (−5–59)	0.173	33.3 (−22.2–85.5)	0.711	0 (0–3)	0.171
	**non-MMC (n = 33)**	11 (8–22)		5 (−1–24)		31.6 (−7.69–70.6)		0 (0–2)	
**12 months**	**MMC (n = 38)**	14 (6–30)	0.812	6 (−12–64)	0.009	30.0 (−66.7–84.2)	0.022	0 (0–2)	0.566
	**non-MMC (n = 32)**	14 (9–28)		3 (−7–20)		15.8 (−35.7–67.9)		0 (0–2)	

**IOP**: intraocular pressure.

**Table 4:** Complications after Deep Sclerectomy.

	Complication	MMC Group n = 44 Eyes	non-MMC Group n = 34 Eyes	*p*-Value
**Perioperative**	Trabeculodescemet microperforation	3 (6.8%)	2 (5.9%)	1
**Early postoperative** (1 day–1 month after surgery)	Hypotony	18 (40.9%)	12 (35.3%)	0.646
	Anterior chamber shallowing	1 (2.3%)	0 (0%)	1
	Choroidal detachment	0 (0%)	6 (17.6%)	0.005
	Hyphema	7 (15.9%)	2 (5.9%)	0.285
	Severe bleb hyperemia	1 (2.3%)	0 (0%)	1
	Bleb leakage	1 (2.3%)	0 (0%)	1
	Iris incarceration	3 (6.8%)	0 (0%)	0.253
	Tenon’s cyst	1 (2.3%)	0 (0%)	1
**Late postoperative** (6 months–12 months after surgery)	Hypotonic maculopathy	0 (0%)	1 (2.9%)	0.436
	Spontaneous hyphema	0 (0%)	1 (2.9%)	0.436
	Overfiltering bleb	1 (2.3%)	0 (0%)	1
**Overall**	**-**	**36**	**24**	**0.286**

Abbreviation: MMC, mitomycin C.

## Data Availability

The datasets generated and/or analyzed during the current study are available from the corresponding author on reasonable request.

## Abbreviations

The following abbreviations are used in this manuscript:

DS	Deep Sclerectomy
EC	Esnoper Clip
GP	Goniopuncture
HEMA	Hydroxyethyl methacrylate
IOP	Intraocular pressure
MMC	Mitomycin C
SD	Standard deviation
